# Validation and comparison of Blenkin Taylor Atlas and the London Atlas on an Australian population subset for dental age estimation

**DOI:** 10.1007/s00414-025-03701-0

**Published:** 2026-01-14

**Authors:** Richard Huynh, Selwin G. Samuel, Vennila Anand, Sobia Zafar, Sakher Al-Qahtani

**Affiliations:** 1 School of Dentistry, The University of Queensland, 288 Herston Road, Herston, Brisbane, QLD 4006 Australia; 2 Department of Oral Pathology and Microbiology, Saveetha Institute of Medical and Technical Sciences (SIMATS), Chennai, Tiruvallur India; 3 Consultant epidemiologist and freelance biostatistician, Vernon, CT USA; 4 Department of Paediatric Dentistry and Orthodontics, College of Dentistry, King Saud University, Riyadh, Saudi Arabia

**Keywords:** Dental age estimation, Blenkin Taylor Atlas, London Atlas, Australian population

## Abstract

**Objectives:**

Individual dental age estimation systems provide essential information in law and forensic sciences for the living and deceased. Considering the multi-ethnic population of Australia, it is important to compare the performance of the internationally recognized London Atlas against the Australian-developed Blenkin Taylor age estimation system.

**Methods:**

218 anonymized digital panoramic radiographs of children aged 5–18 years old were reviewed and 200 met inclusion criteria. The qualified radiographs were subjected to both the London Atlas and Blenkin Taylor age estimation systems, and this was compared to the children’s real ages.

**Results:**

The Blenkin Taylor method produced a statistically significant mean underestimation of 0.34 ± 1.44 years, while the London Atlas also produced a statistically significant mean underestimation of 0.96 ± 1.64 years.

**Conclusion:**

Both age estimation systems produced a significantly smaller difference between the estimated and chronological age for females compared to males. Both the London Atlas and Blenkin Taylor are appropriate to apply in an Australian paediatric population for the purposes of forensic age estimation.

## Introduction

The identification of an individual’s age plays a fundamental role in law and forensic science for both the living and the deceased. The legal system commonly dictates an age of criminal responsibility, distinguishing when an individual is considered a minor as opposed to an adult. This can have an entirely different implication for the individual in cases of education, employment, social support, child abuse, marriage and family, migration, and criminal cases such as rape and kidnapping [1, 2].

A positive confirmation of an individual’s identity can be completed via fingerprint, deoxyribonucleic acid (DNA), or dental analysis [3]. Notably, the teeth are highly mineralised and therefore more resistant to environmental insults, such as from nutrition, hormones, and pathology, more so than the skeletal age indicators [4, 5]. Dental age estimation can be scientifically conducted via either biochemical or radiological methods. Aspartic acid racemization measured through gas chromatography applied to dentine tissue appears to be the most accurate biochemical technique [6]. But these aspartic acid racemization produce more accurate results for adults [7]. Other biomarkers found in the dentition reflecting chronological age include mitochondrial DNA mutations [8], telomere shortening [9], and epigenetics [10]. However, these biomarkers have limitations, including sensitivity to postmortem environmental exposures, variation between population groups, being time-consuming, margins of errors upto 10 years in adults, and relying on extracted dental tissue, which would be inappropriately invasive to apply on a living individual [11].

There are two predominant classes of child dental age estimation using radiological means. First, the atlas methodology involves comparing the individual’s dental data to a standardised visual atlas of tooth formation and/or eruption [2, 11]. One of the most well-known and first atlases is the Schour and Massler, which noted 21 diagrams from 4 months old to 21 years old [12]. Second, the incremental staging methodology, which assigns scores based on statistically analysed stages of dental development [2, 11]. One of the most popular incremental scoring systems is the Demirjian system [2], based on the mineralisation of seven individual permanent mandibular teeth [13].

In 2012, Blenkin and Taylor developed an atlas [14] (herein referred to as ‘Blenkin Taylor’) by re-interpreting the ages in the Ubelaker update of the original Schour and Massler’s Atlas [15], using data derived from an Australian population. Each of the diagrams in the Ubelaker chart was rated according to the Demirjian incremental scoring system as individual cases and converted to age estimates as previously applied by Blenkin [13].

Another atlas system applied to panoramic radiographs is the London Atlas of Tooth Development (herein referred to as the ‘London Atlas’). The London Atlas was developed by applying a combination of the Moorrees incremental scoring system to evaluate the stage of tooth formation and the modified Bengston’s stages to assess the alveolar eruption status on a sample of 704 of mixed British and Bangladesh ethnicity [16]. When compared to other popular dental age estimation systems, the London Atlas has been found to produce more statistically significant correct age estimations than the systems by Schour and Massler, Ubelaker, and Cameriere [12, 17]. Since publication in 2010, the London Atlas has been validated in various ethnic populations, including Portuguese [18], Iranian [19], Hispanic [20], Indian [21], and Saudi Arabian [22], with a recent meta-analysis study showing a standardized mean age difference of 0.02 years [23].

The modified Demirjian system by Blenkin and Evans has been validated [24], which was used to re-interpret the ages on Ubelaker’s Atlas diagrams by Blenkin and Taylor [14]. The Blenkin Taylor Atlas, as well as the London Atlas, has yet to be validated within a wide Australian population.

Therefore, we embarked on a study consisting of two aims. The first aim is to test the performance of the Blenkin Taylor and the London Atlas within an Australian population subset in Queensland. The second aim is to determine whether there is a difference in performance between the Blenkin Taylor and the London Atlas. The three null hypotheses are that (1) there would be no difference between the age estimates by Blenkin Taylor or the London Atlas compared to the chronological age, (2) there would be no difference between the age estimates between males and females when using Blenkin Taylor or the London Atlas compared to the chronological age and (3) there would be no difference between the accuracy of either methodologies.

## Materials and methods

### Ethical approval

Ethical approval was granted by The University of Queensland’s Institutional Human Research Ethics Committee (Approval Number: 2019000998). Only individuals who consented to the use of their radiographic records to be used for research purposes were included.

### Sample selection and size

A retrospective cross-sectional study was planned after the accrual of 218 digital panoramic radiographs of male and female patients aged between 5 and 18 years old from the archival records of the School of Dentistry at The University of Queensland.

### Selection criteria

The samples in this study had the following inclusion criteria:

1. Australian patients.
2. Individuals aged between 5 and 18 years.

The exclusion criteria were:

1. Poor quality radiographs: overexposure, overlap of structures, and presence of artifacts in the region of interest.
2. The presence of any systemic diseases or developmental conditions.
3. Developmental anomalies, but not limited to, Amelogenesis/Dentinogenesis Imperfecta, Taurodontism, Hypodontia, and Hyperdontia.
4. The presence of gross pathology related to the right side of the jaws or teeth.
5. The presence of gross caries and periapical pathosis on the right side of the jaw.
6. The presence of large restorations or crowns on the right side of the jaw.
7. History of tooth extraction(s) on the right side of the jaw.
8. History of maxillofacial trauma and orthodontic treatment.

### Study design

Panoramic radiograph samples were anonymized before inclusion, where the only metadata available was the patient’s date of birth, date on which the radiograph was taken, and sex. The chronological age of each sample was calculated by finding the difference between the date of birth and the date the radiograph was taken after conversion to a decimal age. The chronological age was blinded to evaluators until age estimation was completed. The anonymized radiographs were assessed by one trained evaluator (RH) applying the Blenkin Taylor method and then the London Atlas to produce age estimations of all samples. The same random sample of 10 radiographs was scored by the evaluator (RH) and one other author who is an expert in age estimation (SA), twice, across 2 weeks. The inter- and intra-examiner variations were tested using Cohen’s Kappa.

For the Blenkin Taylor, both the stage of development and the eruption stage of all teeth from the samples were compared with the revised Ubelaker diagrams to produce age estimation for the relevant sex [14]. Where the comparison with the illustrations was ambiguous, an underestimation was favoured. Results were recorded on the template table (Table 1).

**Table 1:** Template table utilised by the evaluator when applying the Blenkin Taylor, and London atlas to sample radiographs for age estimation. The numbers 1–8 indicate the FDI notation for the tooth for the corresponding arch

Anonymized ID	London Atlas																	Blenkin Taylor
	Maxillary								Mandibular								Age Estimation	Age Estimation
	1	2	3	4	5	6	7	8	1	2	3	4	5	6	7	8		

For the London Atlas, the stage of development for each tooth on the right-hand side was entered into the template table (Table 1) and then compared with the pictorial illustrations of the London Atlas to produce age estimation. The London Atlas Software (Queen Mary Innovation Ltd, London, United Kingdom) was not utilised. When the right-hand side tooth was unclear, the corresponding contralateral tooth was examined, provided that it met the exclusion criteria. Where the comparison with the illustrations was unclear, an underestimation was favoured. Sex was not accounted for.

### Statistical analysis

All statistical analyses were conducted with IBM SPSS Statistics for Windows (SPSS version 28.0; IBM Corporation, Armonk, NY, USA). The estimated age by the evaluator was subtracted from the calculated chronological age. An overestimation would reveal a positive value, whereas an underestimation would produce a negative value. Accuracy was defined based on the differences observed between estimated and chronological ages. Accuracy was assessed using the mean difference (bias) and the absolute mean difference between estimated and chronological ages.

Bias was calculated as the mean difference between the estimated and chronological ages for each estimation method using a two-tailed paired t-test across the entire sample, as well as in 12-month age groups (Table 2). The absolute mean difference between the estimated and chronological age was also used to show the accuracy of each estimation method independent of bias. An independent sample t-test was used to compare whether sex influenced the accuracy of either method. Statistical significance was set at a *p*-value < 0.05.

**Table 2:** Sex and age distribution characteristics of the sample

Group	Age range (years old)	Male (*n*)	Female (*n*)	Total (*n*)
5	5.00–5.99	2	3	5
6	6.00–6.99	4	7	11
7	7.00–7.99	10	9	19
8	8.00–8.99	11	9	20
9	9.00–9.99	9	10	19
10	10.00–10.99	3	9	12
11	11.00–11.99	10	10	20
12	12.00–12.99	9	10	19
13	13.00–13.99	9	8	17
14	14.00–14.99	10	8	18
15	15.00–15.99	10	10	20
16	16.00–16.99	10	5	15
17	17.00–17.99	3	2	5
**Total (n)**		100	100	200

## Results

### Reliability

The inter- and intra-observer correlation for the Blenkin Taylor showed a Cohen’s kappa of 0.87 and 0.88, respectively. Meanwhile, the inter- and intra-observer correlation for the London Atlas was 0.89 and 0.9, respectively. These indicate excellent reliability.

### Description of sample

There were 218 digital panoramic radiographs examined, but 18 were excluded due to not being within the required age range of 5–18 years or being missing the requisite metadata, such as date of birth, date of radiograph, or sex. Consequently, 200 radiographs were included for analysis. Table 2 shows the age and sex distribution of the sample used. The mean chronological age of the entire sample was 11.54 years with a standard deviation of 3.27 years.

### Blenkin Taylor Atlas

For the Blenkin Taylor, the mean estimated age of the entire sample was 11.20 years, which produced a mean difference of −0.34 years (SD ± 1.44) and was found to be significant (*p* < 0.05) via a paired t-test (Table 3). Negative values indicate underestimation.

**Table 3:** Accuracy of the Blenkin Taylor expressed by bias and absolute difference for the entire sample

Measure of Accuracy	*N*	Mean	SD	SE	Significance	95% CI Lower	95% CI Upper
Bias	200	−0.3374	1.4445	0.1021	0.0011*	−0.5388	−0.1360
Absolute Difference	200	1.1876	0.8852	0.0626	0.0001*	1.0642	1.3110

N = sample size; SD = Standard deviation; SE = Standard error; CI = Confidence interval; * = p < 0.05

When comparing the accuracy of the Blenkin Taylor across sex, there was a statistically significant difference found with an independent sample t-test (Table 4). The Blenkin Taylor produced a significantly smaller difference between the estimated and chronological age for females compared to males in the bias analysis.

**Table 4:** The accuracy of the Blenkin Taylor atlas across sex expressed by bias and absolute difference

Measure of Accuracy	Sex	*N*	Mean	SD	SE	Significance
Bias	**F**	100	−0.0790	1.5346	0.1535	0.0111*
	**M**	100	−0.5958	1.3055	0.1306	
Absolute Difference	**F**	100	1.2230	0.9222	0.0922	0.5728
	**M**	100	1.1522	0.8498	0.0850	

*F = Female*; M = Male; N = sample size; SD = Standard deviation; SE = Standard error; * = p < 0.05

**Fig. 1:**
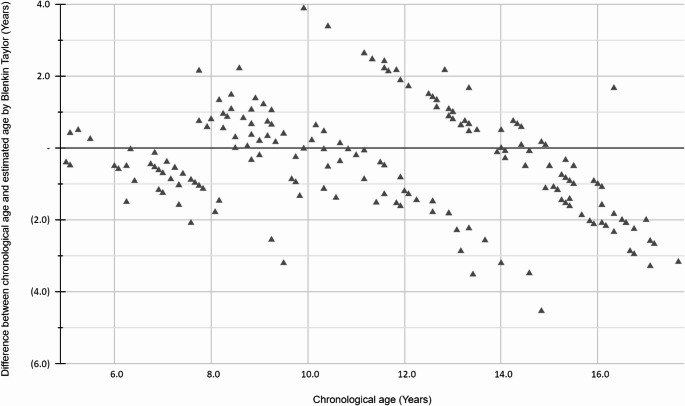
The difference between chronological age and estimated age by the Blenkin Taylor method for each age group

The difference between chronological age and estimated age by the Blenkin Taylor for each chronological age group is illustrated by a Bland-Altman plot (Fig. 1). When applying a paired t-test, bias was found to be statistically significant in Groups 6, 7, 8, 15, 16, and 17, and was under-estimated majorly (Table 5). Among the subgroups, the paired t-test revealed statistical significance in some subgroups. In males, the test showed statistically significant underestimation in Groups 7, 15, 16, and 17 and statistically significant overestimation in Group 8. In females, the test showed statistically significant underestimation in Groups 6, 15 and statistically significant overestimation in Group 11 (Table 5).

**Table 5:** Paired t-test of Blenkin Taylor Atlas entire and across sex expressed by bias

Group	Age subgroup	Mean difference	SD	CI		t	Significance
				Lower	Upper		
Entire	6	−0.630	0.425	−0.916	−0.345	4.917	0.0006*
	7	−0.564	0.971	−1.032	−0.096	2.532	0.0209*
	8	0.552	0.943	0.111	0.993	2.618	0.0169*
	15	−1.110	0.537	−1.361	−0.859	9.247	0.0001*
	16	−1.749	1.089	−2.352	−1.146	6.222	0.0001*
	17	−2.740	0.514	−3.378	−2.102	11.931	0.0003*
Male	7	−0.907	0.368	−1.170	−0.644	7.799	0.0001*
	8	0.564	0.784	0.037	1.090	2.386	0.0382*
	15	−0.933	0.296	−1.145	−0.722	9.969	0.0001*
	16	−2.000	0.258	−2.184	−1.816	24.552	0.0001*
	17	−2.806	0.315	−3.589	−2.022	15.402	0.0042*
Female	6	−0.519	0.262	−0.762	−0.276	5.234	0.0019*
	11	1.693	1.400	0.692	2.695	3.826	0.0041*
	15	−1.287	0.672	−1.767	−0.806	6.056	0.0002*

SD = Standard deviation; CI = Confidence interval; t = paired t-test statistics; * = p < 0.05

**Fig. 2:**
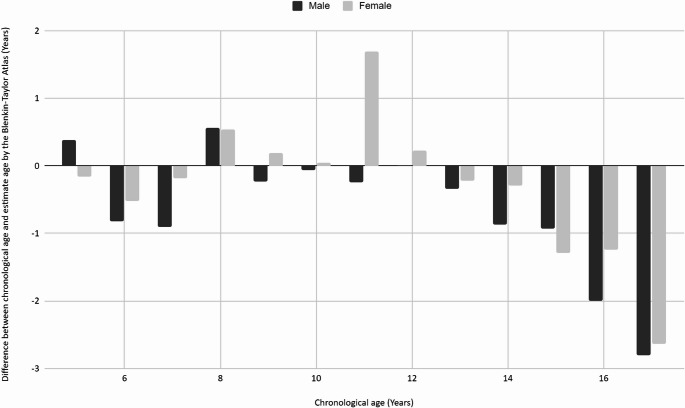
The difference between chronological age and estimated age by the Blenkin Taylor method for each age group subdivided by sex

Figure 2 illustrates the difference between chronological age and estimated age by the Blenkin Taylor for each age group between males and females.

### London Atlas

For the London Atlas, the mean estimated age of the entire sample was 10.58 years, which produced a mean difference of −0.96 years (SD ± 1.64) and was found to be significant (*p* < 0.05) via a paired t-test (Table 6). Negative values indicate underestimation.

**Table 6:** Accuracy of the London Atlas expressed by bias and absolute difference for the entire sample

Measure of Accuracy	*N*	Mean	SD	SE	Significance	95% CI Lower	95% CI Upper
Bias	200	−0.9617	1.6441	0.1163	0.0001*	−1.1909	−0.7324
Absolute Difference	200	1.4050	1.2840	0.0908	0.0001*	1.2260	1.5840

N = sample size; SD = Standard deviation; SE = Standard error; CI = Confidence interval; * = p < 0.05

When comparing the accuracy of the London Atlas across sex, there was a statistically significant difference found when applying an independent sample t-test (Table 7), meaning that the London Atlas produced a significantly smaller difference between the estimated and chronological age for females compared to males for both bias analysis and absolute difference.

**Table 7:** The accuracy of the London Atlas across sex expressed by bias and absolute difference

Measure of Accuracy	Sex	*N*	Mean	SD	SE	Significance
Bias	**F**	100	−0.5650	1.5732	0.1573	0.0006*
	**M**	100	−1.3583	1.6250	0.1625	
Absolute Difference	**F**	100	1.1800	1.1793	0.1179	0.0128*
	**M**	100	1.6300	1.3494	0.1349	

*F = Female*; M = Male; N = sample size; SD = Standard deviation; SE = Standard error; * = p < 0.05

When applying a paired t-test, bias was found to be a statistically significant overestimation in Groups 5, 8, and 10 and a statistically significant underestimation in Groups 12, 13, 14, 15, 16, and 17 (Table 8). Among the subgroups, the paired t-test revealed statistical significance in some subgroups. In males, the test showed statistically significant underestimation among Groups 11, 12, 13, 14, 15, 16, and 17. In females, the test showed statistically significant overestimation in groups 6, 8, 10, and statistically significant underestimation in groups 12, 14, 15, 16, and 17 (Table 8).

**Table 8:** Paired t-test of the London atlas entire and across sex expressed by bias

Group	Age subgroup	Mean difference	SD	CI		t	Significance
				Lower	Upper		
Entire	5	0.717	0.481	0.120	1.314	3.332	0.0290*
	8	0.492	0.794	0.120	0.863	2.770	0.0122*
	10	0.486	0.515	0.159	0.813	3.272	0.0074*
	12	−1.096	0.664	−1.416	−0.776	7.199	0.0001*
	13	−1.441	1.056	−1.984	−0.898	5.625	0.0001*
	14	−2.046	1.289	−2.687	−1.405	6.734	0.0001*
	15	−2.650	1.084	−3.157	−2.143	10.932	0.0001*
	16	−3.389	1.259	−4.086	−2.691	10.422	0.0001*
	17	−4.700	0.267	−5.032	−4.368	39.296	0.0001*
Male	11	−0.608	0.801	−1.182	−0.035	2.401	0.0398*
	12	−1.426	0.704	−1.967	−0.885	6.077	0.0003*
	13	−1.898	0.694	−2.432	−1.364	8.200	0.0001*
	14	−2.508	1.250	−3.403	−1.614	6.346	0.0001*
	15	−2.833	1.125	−3.638	−2.029	7.965	0.0001*
	16	−3.250	1.261	−4.152	−2.348	8.152	0.0001*
	17	−4.806	0.315	−5.589	−4.022	26.382	0.0014*
Female	6	0.738	0.552	0.228	1.248	3.540	0.0122*
	8	0.648	0.816	0.021	1.276	2.382	0.0444*
	10	0.602	0.532	0.193	1.010	3.396	0.0094*
	12	−0.800	0.485	−1.147	−0.453	5.220	0.0005*
	14	−1.469	1.158	−2.437	−0.501	3.587	0.0089*
	15	−2.467	1.068	−3.231	−1.703	7.303	0.0001*
	16	−3.667	1.353	−5.346	−1.987	6.061	0.0037*
	17	−4.542	0.059	−5.071	−4.012	109.000	0.0058*

SD = Standard deviation; CI = Confidence interval; t = paired t-test statistics; * = p < 0.05

**Fig. 3:**
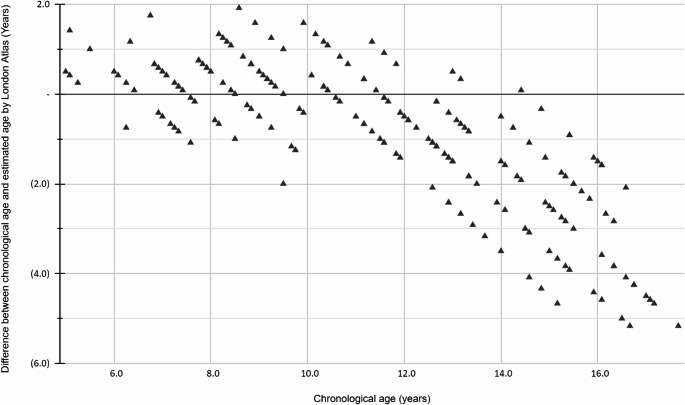
The difference between chronological age and estimated age by the London Atlas for each age group

Figure 3 illustrates the difference between chronological age and estimated age by the London Atlas for each chronological age group.

**Fig. 4:**
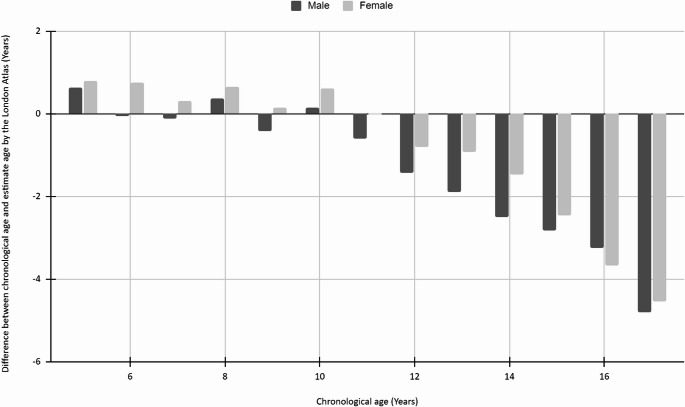
The difference between chronological age and estimated age by the Blenkin Taylor method for each age group subdivided by sex

Figure 4 The difference between chronological age and estimated age by the Blenkin Taylor method for each age group subdivided by sex.

## Discussion

Both the Blenkin Taylor Atlas and the London Atlas had yet to be validated within a wide Australian population. Hence, this study had two predominant aims: (1) to test the performance of the Blenkin Taylor and the London Atlas within a population of Queensland, Australia; and (2) to determine whether there is a difference in the performance between the Blenkin Taylor and the London Atlas. The accuracy of the Blenkin Taylor and London Atlas methods was tested on a sample of 200 radiographs (100 male and 100 female) of individuals aged 5–18 years, and the influence of chronological age and sex was determined on their accuracy.

### Entire sample

The mean chronological age of the entire sample was 11.54 (± 3.27) years. There was a statistically significant underestimation for both the Blenkin Taylor (−0.34 ± 1.44 years) and the London Atlas (−0.96 ± 1.64 years). Thus, the null hypothesis that there would be no difference between the age estimates by Blenkin Taylor or the London Atlas compared to the chronological age was rejected, and both the Blenkin Taylor and London Atlas produced significant underestimations of the sample In contrast, a study testing the accuracy of the Blenkin Taylor on an Indonesian population found an overestimation of 0.051 (CI: −0.393-0.496) years, but this was not statistically significant, potentially because of a low sample size (*n* = 34) [25] On the other hand, the literature appears divided on whether the London Atlas tends to underestimate or overestimate the study populations’ age. Similar to this Australian study, a Saudi study found there was a statistically significant underestimation [17], but was in contrast to another Australian study [26] and Turkish study [27], which found a statistically significant overestimation. Some studies failed to achieve statistical significance [21, 28] In the present study, the underestimation observed in both the Blenkin Taylor and the London Atlas may be the result of underestimation in cases of ambiguity. Although this may introduce a systematic bias, the minimum age concept places the legal onus on determining whether the individual has reached the age of legal majority with a degree of certainty; otherwise, that individual is afforded the privileges of being a minor [29]. Therefore, the conservative underestimation adopted in this study is appropriate to establish, with reasonable certainty, an individual’s minimum age rather than their maximum age.

### Sex

Both the Blenkin Taylor and the London Atlas produced a statistically significant difference between males and females, and thus the null hypothesis was rejected. Both estimation methods produced a smaller difference between the estimated and chronological age for females compared to males. One New Zealand study found that when applying a percentage ‘best fit’ analysis to each age category, the Blenkin Taylor was more accurate for males than females, but their comparative difference between sexes was not tested for significance [30] On the flipside, the London Atlas produced a smaller difference between the estimated and chronological age for females compared to males, but none of these studies achieved statistical significance [17, 21]. In contrast, other studies demonstrated that the London Atlas was more likely to have a greater overestimation of females compared to males [27, 31–34] The inconsistency in the literature can be explained by the fact that these statistics were produced based only on sex and did not account for age. Although females generally develop teeth earlier than males [14], the start and rate of development may be different for each age group.

### Age

In examining the influence of age on the estimation methods, Blenkin Taylor found there was statistical significance achieved only in the extremities of the age spectrum (Groups 6, 7, 8, and 15, 16, 17), which were all underestimated. Meanwhile, the London Atlas found there was a statistically significant overestimation in younger age groups (Groups 5, 8, 10) and underestimation in older age groups (Groups 12, 13, 14, 15, 16, 17) Neither of the two studies testing the accuracy of the Blenkin Taylor reported on any differences between ages without accounting for sex [25, 30] This study’s findings with the London Atlas were very similar to a study on a Saudi population, where there was also a non-statistically significant over-estimation in most younger age groups (6–9 years old) and statistically significant overestimation in all older age groups (10–15 years old) [17]. A Colombian study also found a statistically significant underestimation in even older age groups (21–23 years old) [31]. Alshihri et al. also found that the London Atlas was least accurate for samples aged 16 years and older [33]. The inaccuracy within the older age groups is likely because the London Atlas relies on the third molars, which have great variability in development and eruption between individuals [34–36]. On the other hand, an Indian study found statistically significant differences on dental age estimation between normal and disabled children (deaf & dumb) [37]. This finding suggests that Australian children with disabilities may also have deviated eruption patterns.

### Age and sex

Statistical significance was found in the Blenkin Taylor method for males in Groups 7, 8, 15, 16, and 17, and females in Groups 6, 11, 15. For the London Atlas, statistical significance was found for males in Groups 11, 12, 13, 14, 15, 16, and 17, and females in Groups 6, 8, 10, 12, 14, 15, 16, and 17. The null hypothesis, stating that there would be no difference between the age estimates for males and females, was rejected. The only other study to have tested both the accuracy of the Blenkin Taylor and London Atlas on the same population was conducted in New Zealand [30]. It was found that the Blenkin Taylor produced statistically significant results for males aged 5.00–5.99, 6.00–6.99, 10.00–10.99, 13.00–13.99, and females aged 5.00–5.99, 6.00–6.99, 9.00–9.99, 10.00–10.99, and 14.00–14.99. Meanwhile, statistical significance was achieved with the London Atlas for males aged 10.00–10.99, 11.00–11.99, 13.00–13.99, 14.00–14.99, 15.00–15.99, and females aged 15.00–15.99 only. Further studies examining the combined influence of sex and age on the accuracy of the London Atlas produced no consistent pattern of inaccuracy [17, 21, 27, 33]. Although we tried to represent an equal number of men and women across the entire sample, a weakness in the analysis is a lack of homogeneity of men and women for each age group, which can affect the statistical outcome [38]. Nonetheless, for both the Blenkin Taylor and London Atlas, accuracy was more influenced by age group rather than sex, especially in the older age groups, where both estimation methods relied more on the highly variable development and eruption of the third molars.

### Overall accuracy

Overall, the Blenkin Taylor produced statistically significant smaller bias and absolute mean difference between the estimated age and chronological age compared to the London Atlas. Thus, the null hypothesis was rejected as the Blenkin Taylor was more accurate than the London Atlas, which is consistent with the study applying both methodologies on a New Zealand population [30]. Although both age estimation systems tested provided a conservative underestimation of age, aligning with the minimum age concept, these are both radiological dental age estimation systems. Like any other forensic age estimation method, the overall accuracy of the age estimation process can be improved by using a combination of methods, such as clinical dental examination, hand x-ray, and clavicle assessment [29].

## Limitations and future directions

We acknowledge that the sample size and the sub-groups were under-represented because this was a retrospective cross-sectional study, which limited the number of eligible cases available for inclusion. The main limitations were the small sample size, unequal age distribution, and unequal distribution of sex within the age subgroups. Indeed, Australia is one of the most culturally diversified countries, with the 2021 census reporting that 27.6% of the Australians were born overseas and that ancestry is widely varied, including English, Australian, Irish, Scottish, Chinese, Indian and many other backgrounds. Much of the current population is descended from migrants, and the broad ethnic diversity reflects shifting migration patterns over time [39]. Thus, another limitation of this study was the inability to stratify the present study’s results or analysis by ethnic population subgroups, even though age estimation systems tend to be based on a mono-ethnic population [14]. This ethnic heterogeneity may influence dental development, tooth eruption, and morphological variation. Therefore, applying atlases obtained from relatively homogeneous populations may affect accuracy. This highlights the need to test and compare the performance of the Blenkin Taylor and the London Atlas within a multi-ethnic population. Finally, all radiographs in this study were obtained from a single source. Future studies should aim to include larger samples from multiple centres and a more balanced distribution of ethnicities and age subgroups to improve accuracy and generalisability of the findings.

## Conclusion

This is the first study to compare the Blenkin Taylor with the London Atlas in Australia. The Blenkin Taylor produced a significantly smaller bias and absolute mean difference between the estimated age and chronological age, and thus was deemed more accurate than the London Atlas. This study found that there was a significant difference between sex and age groups both in isolation and in combination. However, a greater paediatric age is likely to influence the accuracy of both the Blenkin Taylor and London Atlas, as both age estimation methods rely on the highly variable development and eruption of the third molars. Nevertheless, given the ethnic diversity of Australia, a consistency in the accuracy of ages of the individuals involved in the study demonstrates the validity of the Blenkin Taylor and the London Atlas comparison methods, not only for the Australian population but also potentially for many other multi-ethnic groups worldwide.

## Declarations

### In memoriam

Sakher Al-Qahtani, who sadly passed away a few months before the submission of this manuscript, made lasting contributions to forensic odontology, particularly in dental age estimation. His work will continue to guide and influence future generations. He will be remembered with deep respect and admiration.
